# The COVID-19 Pandemic: A Family Affair

**DOI:** 10.1177/1074840720920883

**Published:** 2020-05-19

A new viral illness called coronavirus disease 2019 (COVID-19) is currently spreading throughout the world at an alarming rate (Dong et al., 2020). As family nursing practitioners, educators, and researchers, we work from a guiding assumption that health and “illness is a family affair” (Wright & Bell, 2009, p. ix). Patients, clients, residents, and their families are inextricably connected. The science and practice of Family Nursing is based on a systemic premise offered by Wright and Leahey (2013) that serious illness and life challenges impact the family unit, and reciprocally, the functioning of the family unit (including their structure, development, and function) influences the health and well-being of each family member. This especially holds true for the current coronavirus pandemic which is creating unique hardships and suffering in an alarmingly large number of patients and their families around the world.

## Impact of COVID-19 on Families

The assumption that disease and its prevention is a family affair is manifested in the full spectrum and scale of the current coronavirus pandemic. Measures that have been taken in many countries to control the spread of the coronavirus are having a disruptive effect on relationships in general and family relationships specifically. Families are reporting loss of community and freedom of movement in response to quarantine/lock down measures. Other tangible losses include income, access to resources, and planned activities or celebrations. Compelling and heartbreaking stories of the challenges and suffering that families are experiencing engulf us. Individuals and families who are most vulnerable are particularly at risk.

### Patients, Clients, and Residents Miss Their Family and Loved Ones

Residents in long-term care facilities miss their partners and children who are no longer allowed to visit because of the COVID-19 policies to contain the spread of the coronavirus. People with intellectual disabilities who live in institutions are upset because their father, mother, brothers, or sisters are no longer allowed to visit, and they often cannot understand why.

Distressing stories abound of patients who have to deal with the news of their COVID-19 diagnosis all by themselves without a family member present and those patients who are admitted to an intensive care unit (ICU) who have to say goodbye to their family in the emergency department not knowing whether they will see each other again. A nurse working at the front line of triage reported, “His family was not allowed to come to the hospital, because they may also be infected. He was alone and couldn’t say goodbye.”

### Families are Concerned About Their Loved Ones

Many of these patients, clients, and residents are members of families who miss their loved ones and who are worried. Mothers, fathers, and other family members of children receiving psychiatric care report being unable visit their child for an extended period of time and are afraid their child will become ill from the coronavirus. Families of very seriously ill and dying patients are not allowed to visit their loved one and may not be able to say a final goodbye.

### Families and Family Relationships Are Under Pressure

The lockdown/quarantine measures instituted in many countries have also invited vulnerability and risk within families. Schools are being closed which leads to distress in many families not accustomed to being so closely confined for a long time period. Moreover, as a result of the COVID-19 crisis, much, if not all, of the support given to families who provide long-term care for an ill parent, partner, or child is lost. Families with a child who requires specialized care and guidance now have to care for their child 24 hr a day without the outside guidance provided by medical nursery, daycare, or special education services. Families who care for a father, mother, or partner with dementia or other serious illness now have to manage without day care or daytime activity. School closures have created a family environment where children are rarely allowed to leave the house and are confronted by the vulnerabilities of a family member’s addiction, aggression, and violence. Children of divorced co-parents are suddenly being refused alternating parental care because one of the parents now works from home and cannot provide child care. All of these families and their interrelationships are often under great pressure as a result of the stresses created by coronavirus pandemic.

## The Urgent Need for Family Nursing Now and in the Aftermath of COVID-19

Health care professionals, including nurses and doctors, are also going through a very intensive and perhaps traumatic time. As a nurse working in the ICU reported, “Many people die without family present. The sorrow that comes with it hurts the nurses mentally.” It is encouraging to see how innovative and creative many nurses are becoming during this pandemic as they find ways to involve families. Despite being committed to the care of the ill person, they assure families that their family member is being cared for and will not die alone. They are sometimes able to connect family members to each other using new technology. Mobile phone or video conference calls made by the nurse allow family members to “see” the patient in the ICU or in the nursing home.

Our concerns also focus on the long-term implications for patients and their families; how will they cope once the coronavirus is under control? How will they be able to resume normal life again? Individuals and families are often flexible and resilient (Walsh, 2016) and many will likely be able to process the experiences of this pandemic and resume their lives. However, there will also be long-term mental and physical health consequences or even permanent damage.

For example, how will patients and families recover after a long period of intensive care? Research has documented that many patients experience many physical and psychological problems after such a long period of ventilation, even after discharge to their home environment (Rawal et al., 2017). We also know that family members of these patients also suffer greater levels of depression and anxiety (Davidson et al., 2012).

We also hold our hearts out for families who have lost someone without being able to say goodbye and without being able to be present in the final dying phase of their loved one. How will we assist these families to cope with their loss and complex grief?

Our concerns also go out to the health care professionals, especially nurses and doctors, in the aftermath of this COVID-19 crisis. They too will need support in recovering from their suffering and distress.

The good news is that there is compelling evidence that our family nursing assessment and intervention skills can assist families to heal. This pandemic makes us more deeply aware of the important role of family in the lives of patients, clients, and residents. We anticipate that this increased awareness will help us advocate even more strongly for the importance of family nursing during and after this coronavirus crisis. Rightfully, a great amount of money and resources are now being spent to fight the COVID-19 virus. But lives saved must also be lives worth living afterwards.

We believe that family nursing knowledge, developed over the last 40 years, unequivocally offers the necessary skills to help families recover and heal from the expected and unexpected long-term consequences of this pandemic (International, Family, Nursing, & Association, 2015, 2017). The aftermath of COVID-19 calls for a substantial increase in the resources needed to (a) enable nurses to assess and intervene with families in need of support, (b) educate nurses to offer highly skilled family nursing care, and (c) conduct research which provides compelling evidence that family nursing assessment and intervention is effective in addressing illness suffering (Wright, 2017; Wright & Bell, 2009) and optimizing family health. Family nursing has never been more relevant or more urgently needed than now.

This Guest Editorial has been written by members of the **FAM**ily health in **E**urope-**R**esearch in **N**ursing (FAME-RN) group:

Marie Louise A. Luttik, PhD, RN, Professor in Family Nursing & Family Care, Hanze University of Applied Sciences, Research Group Nursing Diagnostics, Groningen, the Netherlands. Email: m.l.a.luttik@pl.hanze.nl ORCID: https://orcid.org/0000-0002-7853-9773

Romy Mahrer-Imhof, PhD, Professor for Family-Centered Care, Nursing Science & Care Limited, Winterthur, Switzerland; Visiting Professor, Department of Clinical Research, University of Southern Denmark, Denmark. Email: romy.mahrer@ns-c.ch ORCID: https://orcid.org/0000-0002-8587-3817

Cristina García-Vivar, PhD, RN, Senior Associate Professor, Faculty of Health Sciences, Public University of Navarre; Researcher, IdiSNA, Navarra Institute for Health Research, Spain. Email: cristina.garciavivar@unavarra.es ORCID: https://orcid.org/0000-0002-6022-559X

Anne Brødsgaard, PhD, RN, Senior Researcher, Department of Pediatrics and Adolescent Medicine, Copenhagen University Hospital Amager Hvidovre; The Capital Region of Denmark & Section for Nursing, Department of Public Health; The Faculty of Health, Aarhus University, Denmark. Email: anne.broedsgaard.madsen@regionh.dk ORCID: https://orcid.org/0000-0002-5029-9480

Karin B. Dieperink, PhD, RN, Associate Professor, Head of research, Family focused health care research Center (FaCe) and Vice Head, Department of Clinical Research, University of Southern Denmark; Department of Oncology, Odense University Hospital, Denmark. Email: Karin.dieperink@rsyd.dk ORCID: https://orcid.org/0000-0003-4766-3242

Lorenz Imhof, PhD, Professor for Community-Based Care, Nursing Science & Care Limited, Winterthur, Switzerland. Email: lorenz.imhof@ns-c.ch ORCID: https://orcid.org/0000-0001-8441-3598

Birte Østergaard, PhD, Associate Professor, Department of Clinical Research, University of Southern Denmark, Denmark. Email: boestergaard@health.sdu.dk ORCID: https://orcid.org/0000-0002-9094-8123

Erla Kolbrun Svavarsdottir, RN, PhD, FAAN, Professor, School of Health Sciences, Faculty of Nursing, University of Iceland, Iceland. Email: eks@hi.is ORCID: https://orcid.org/0000-0003-1284-1088

Hanne Konradsen, PhD, Professor, Herlev and Gentofte Hospital, Department of Gastroenterology, Denmark, Department of Clinical Medicine, Faculty of Health and Medical Sciences, University of Copenhagen, Denmark; Associate Professor, Department of Neurobiology, Care Sciences and Society, NVS, Karolinska Instituttet, Sweden. Email: hanne.konradsen@regionh.dk ORCID: https://orcid.org/0000-0002-7477-125
